# Impact of Starch-Rich Food Matrices on Black Rice Anthocyanin Accessibility and Carbohydrate Digestibility

**DOI:** 10.3390/foods12040880

**Published:** 2023-02-18

**Authors:** Sean Jun Leong Ou, Amanda Simin Fu, Mei Hui Liu

**Affiliations:** 1 Department of Food Science and Technology, National University of Singapore, 3 Science Drive 3, Singapore 117543, Singapore; 2 Department of Medicine, Yong Loo Lin School of Medicine, National University of Singapore, 14 Medical Drive, Singapore 117599, Singapore

**Keywords:** polyphenol, functional food, fortification, coingestion, starch digestibility

## Abstract

Anthocyanins reduce starch digestibility via carbohydrase-inhibitory pathways, but food matrix effects during digestion may also influence its enzymatic function. Understanding anthocyanin-food matrix interactions is significant as the efficiency of carbohydrase inhibition relies on anthocyanin accessibility during digestion. Therefore, we aimed to evaluate the influence of food matrices on black rice anthocyanin accessibility in relation to starch digestibility in common settings of anthocyanin consumption—its co-ingestion with food, and consumption of fortified food. Our findings indicate that black rice anthocyanin extracts (BRAE) had reduced intestinal digestibility of bread to a larger extent for the co-digestion of BRAE with bread (39.3%) (4CO), than BRAE-fortified bread (25.9%) (4FO). Overall anthocyanin accessibility was about 5% greater from the co-digestion with bread than fortified bread across all digestion phases. Differences in anthocyanin accessibility were also noted with changes to gastrointestinal pH and food matrix compositions—with up to 10.1% (oral to gastric) and 73.4% (gastric to intestinal) reductions in accessibility with pH changes, and 3.4% greater accessibility in protein matrices than starch matrices. Our findings demonstrate that the modulation of starch digestibility by anthocyanin is a combined result of its accessibility, food matrix composition, and gastrointestinal conditions.

## 1. Introduction

The shift in diets to one rich in polyphenols has been advocated as a dietary strategy against the pathogenesis and regression of metabolic disorders [1,2]. Anthocyanins are one of such phytochemicals that have been known for its beneficial effects in mitigating postprandial glycaemic control through the suppression of carbohydrate digestion, suggesting its use as natural substitutes to anti-diabetic drugs for improved glycaemic control. In comparison to popular sources of anthocyanins such as berries, black rice has garnered much interest and distinguishes itself from anthocyanin sources via its nutritive properties and health benefits when consumed regularly as a staple food. Its major anthocyanins comprise cyanidin-3-glucoside (C3G), cyanidin-3-rutinoside (C3R), peonidin-3-glucoside (P3G), and cyanidin-3,5-diglucoside (C35D) [3]; which have been demonstrated in-vitro to be potent inhibitors of amylases and glucosidases [4]. Black rice and its extracts have thus been used to enhance the nutritive and functional properties in starch-rich food applications, often to limit the digestion of starch [5,6].

The efficacy of carbohydrase inhibition is, however, limited by the availability of free anthocyanins from food matrices under the physiological conditions of digestion. In nutrient metabolism, accessibility is generally referred to as the fraction of nutrients liberated from the food matrix into the gastrointestinal tract and are available for absorption [7]. Nutrients are usually consumed either in their pure form or as a component associated with food matrices, the latter of which is often characterized by reduced accessibility. When used in food enrichment, polyphenol accessibility may be affected when food matrices entrap and mitigate the release of the polyphenol [8]. On the other hand, when ingested with a meal, molecular interactions between polyphenols and meal components may modify the availability of free polyphenols [8]. The pH changes during gastrointestinal digestion also modulate the chemical stability of anthocyanins, which influences the eventual availability of anthocyanins for absorption [2]. It is thus worth emphasizing that the stability, extent to which anthocyanins are released from their food matrices, and interactions with other dietary components are major contributors to the eventual bioactivity of anthocyanins.

Limited studies have investigated the gastrointestinal accessibility of anthocyanins in foods [2,9,10] or have examined the influence of food matrices on anthocyanin accessibility [9,11,12]. In addition, no studies have evaluated the differences in accessibility of anthocyanins between their co-ingestion with foods and when used in food fortification, especially when these are two common ways anthocyanins can be ingested. Notably, there was also a lack of evidence relating the food matrices and anthocyanin accessibility to the suppression of starch digestion. Therefore, the objective of this study is to investigate the interactions of black rice anthocyanins and starch-rich food matrices on anthocyanin accessibility during each phase of gastrointestinal digestion. Using wheat bread as a vehicle for anthocyanin delivery, we examined the effects of starch-rich food matrices on black rice anthocyanin accessibility in relation to the gastrointestinal digestibility of starch, in two common settings of anthocyanin ingestion (i.e., co-ingestion of anthocyanins and anthocyanin fortification).

## 2. Materials and Methods

### 2.1. Materials

Materials used for the preparation of bread included bread flour (13% protein), vegetable shortening, cane sugar, salt, and dry active yeast, which were purchased from a local supermarket. BRAE used in the fortification of bread was (24.1% of total anthocyanins) obtained from Shaanxi Bolin Biotechnology Co Ltd. (Shaanxi, China).

Materials used for in-vitro experimentation included wheat starch (S5127) and gluten (G5004), porcine pancreatic α-amylase (A3176), porcine pancreatic pepsin (P6887), porcine pancreatin (P7545), and α-amyloglucosidase from *Aspergillus niger* (A7095), which were obtained from Merck Pte Ltd. (Darmstadt, Germany). The D-glucose hexokinase assay kit (K-GLUHK) was obtained from Megazyme Inc. (Wicklow, Ireland).

### 2.2. Bread Sample Preparation

The bread was decided upon as a potential delivery vehicle for anthocyanins as bakery products are among the most commonly consumed foods rich in rapidly digestible carbohydrates [13]. Bread samples in this study (CON and 4FO) were prepared according to Ou, Yu, Zhou, and Liu [5]. Bread flour was fortified with BRAE at 0 g and 4 g of extract per 100 g of flour for the preparation of CON and 4FO bread samples, respectively. Briefly, flour, shortening, yeast, salt, water, sugar, and BRAE were mixed for 6 min in an electric mixer. The resulting dough was divided and hand-molded into 100 g boules after leaving it to rest for 15 min. Dough boules were held in baking molds of 10 × 2 cm (diameter × depth) while being fermented (40 °C, 85% relative humidity, 70 min), baked in an oven (200 °C, 8 min), then cooled for 1 h at room temperature. Fresh breadcrumbs were blast frozen for 4 h before lyophilisation for 72 h. Lyophilised samples were stored in a desiccator at room temperature prior to further analyses. Fresh samples were used for all replicate analyses.

### 2.3. Evaluation of Starch Digestibility

A simulated gastrointestinal digestion was conducted to assess the impact of BRAE fortification on bread digestibility, according to the standardised methods developed by Brodkorb et al. [14]. Additionally, to assess the digestibility-modulating efficacy of BRAE under the influence of matrix effects from wheat bread, BRAE was co-digested with CON (henceforth referred to as 4CO) to simulate the ingestion of bread with the extract, separately. The mass of BRAE used in 4CO was such that the total anthocyanin content in both experimental models (4CO and 4FO) were equivalent. Preparation of the required simulated digestion fluids and enzyme solutions is provided in Appendix A (Table A1 and Table A2).

To simulate oral digestion, the mastication process was first replicated via a brief homogenisation (10,000 rpm) of breadcrumbs and distilled water (1:4 *w/v*) to reduce the viscosity of the oral bolus. Simulated salivary fluid (SSF), α-amylase solution (75 U/mL), and 0.3 M CaCl_2_ (1.5 mM) were mixed with 2 g of homogenised samples. The mixtures were topped up to volume with distilled water and incubated for 2 min at 37 °C in a water bath with agitation. Aliquots of 100 μL were quenched in 900 μL of ethanol after incubation.

To simulate gastric digestion, simulated gastric fluids (SGF), pepsin solution (2000 U/mL), and 0.3 M CaCl_2_ (0.15 mM) were added to the oral digesta and adjusted to pH 3.0. The mixtures were topped up to volume with distilled water and incubated for 2 h at 37 °C in a water bath with agitation. Aliquots of 100 μL at 0, 15, 30, 45, 60, 90, and 120 min were quenched in 900 μL of ethanol.

For intestinal digestion, simulated intestinal fluids (SIF), α-amyloglucosidase (1.3 U/mL), pancreatin (100 U/mL, determined from trypsin activity), and 0.3 M CaCl_2_ (0.6 mM) were added to the gastric digesta and adjusted to pH 7.0. The mixtures were topped up to volume with distilled water and incubated for 4 h at 37 °C in a water bath with agitation. Aliquots of 100 μL at 0, 2, 4, 6, 8, 10, 20, 30, 40, 50, 60, 90, 120, 150, 180, 210, and 240 min were quenched in 900 μL of ethanol.

Quenched aliquots were centrifuged for 10 min at 15,000 g. The resulting supernatant was used to quantify the glucose released according to the Megazyme D-glucose hexokinase assay kit (K-GLUHK). Starch hydrolysis data was fitted into the first order equation Equation (1).

(1) $$C_{t}=C_{0}+\left(C_{\infty }-C_{0}\right)\left(1-\exp\left(-kt\right)\right)$$

where $C_{t}$ is the amount of glucose released at time t (minutes), $C_{\infty }$ is the equilibrium percentage of glucose released after 240 min, and k is the kinetic constant.

Using the trapezoid rule, area under curves (AUC) was determined from the fitted hydrolysis plots generated from Equation (1).

### 2.4. Evaluation of Anthocyanin Accessibility

To determine the effects of food matrices and digestive phases on the gastrointestinal accessibility of anthocyanins, the anthocyanin content in each digestion phase was quantified for both 4FO and 4CO samples. Aliquots of digesta from the end of each simulated digestion phase (oral, gastric, and intestinal) were centrifuged at 8000× *g* at 20 °C for 10 min to quench the mixtures. The supernatants were collected for anthocyanin content determination, as described in Section 2.5. The accessibility of anthocyanins was expressed as a ratio of the anthocyanin content in the supernatant relative to that in crude BRAE.

To evaluate the effects of interactions between anthocyanins and digestion matrix constituents on anthocyanin accessibility, the anthocyanin content of mixtures comprising BRAE with starch, gluten, or enzymes was quantified. Briefly, different distributions of wheat starch and gluten were mixed with water (1:4 *w/w*) and heated at 100 °C for 5 min. The mixtures were allowed to cool to room temperature, and to allow for starch gelatinization. Next, 2 g of each mixture was mixed with 80 mg of BRAE. Simulated fluids without digestive enzymes, corresponding to the volumes used in Section 2.3, were added to the BRAE-mixture. Similarly, 80 mg of BRAE was mixed with simulated fluids with digestive enzymes, corresponding to the volumes used in Section 2.3. The BRAE-mixtures were incubated in a 37 °C water bath for 10 min to allow for interactions between anthocyanins and starch, gluten, or enzymes. The supernatants were collected for anthocyanin content determination, as described in Section 2.5.

### 2.5. Determination of Anthocyanin Content

The extraction of anthocyanins from BRAE and 4FO was performed according to the methods by Ou, Yu, Zhou, and Liu [5]. BRAE (1.0 g) and lyophilized 4FO bread (5.0 g) samples were macerated four times, each for 30 min in 10 mL of trifluoroacetic acid (0.01% *v/v*) in methanol using at 200 rpm. After each maceration, the samples were centrifuged for 10 min (8000× *g*, 20 °C). The supernatants were combined and filtered through a 11 μm pore-sized filter paper and condensed at 40 °C under vacuum. The condensed liquid extracts were topped up with 0.1% formic acid and stored away from light at −20 °C until further analyses. A ×200 and ×10 dilution of condensed extracts from BRAE and lyophilized 4FO samples were performed, followed by filtration through a 0.22 μM PTFE filter, prior to analysis.

Total monomeric anthocyanin was quantified by the pH differential method, according to the AOAC Official Method 2005.02 [15], which calculates anthocyanin content based on their molecular weights and extinction coefficients. With natural matrices often comprising varying mixtures of anthocyanins, anthocyanin concentrations are officially expressed as equivalents of C3G, which is the predominant anthocyanin in nature. Condensed liquid extracts from BRAE and 4FO were diluted in pH 1.0 (0.025 M potassium chloride) and pH 4.5 (0.4 M sodium acetate) buffers. The dilution factors were determined by obtaining an absorbance between 0.2 and 1.4 AU at 520 nm through sample dilution with the pH 1.0 buffer. The samples were read at 520 and 700 nm within 50 min of preparation against a sample blank. Total anthocyanin content (TAC) was expressed in C3G equivalents according to Equation (2)

(2) $$TAC=\left\{\left[{\left(A_{520nm}-A_{700nm}\right)}_{pH\;1.0}-{\left(A_{520nm}-A_{700nm}\right)}_{pH\;4.5}\right]\times MW\times DF\times 10^{3}\right\}/\left(\varepsilon \times 1\right)$$

where molecular weight, MW = 449.2 g/mol for C3G; dilution factor, DF = 20, molar extinction coefficient, ɛ = 26,900 L cm^−1^ mol^−1^ for C3G.

To determine the distribution of major anthocyanins in the extracts and supernatants, samples were analyzed using a reverse-phased C_18_ Sunfire column (250 × 4.6 mm/5 μm; Waters, Wexford, Ireland) on a HPLC system (Shimadzu Prominence, Shimadzu, Kyoto, Japan) connected with a diode array detector (DAD). The injection volume was 50 μL. The flow rate and oven temperature were maintained at 1 mL/min at 25 °C. A gradient elution process was applied (mobile phase A: 5% *v/v* formic acid; mobile phase B: 100% acetonitrile): 0% B for 5 min, 10% B at 20 min, 13% B at 40 min, 20% B at 44 min, 25% B at 50 min, and 100% B at 55 min. Detection of anthocyanins was performed at 520 nm. Identification and quantification of each anthocyanin was based on matching the retention time and peak areas with the external calibration curve of respective standards.

### 2.6. Statistical Analysis

Experiments were carried out in triplicates, with three repeats conducted for each replicate, and expressed in means with standard error of means, unless stated otherwise. Statistical analyses were conducted using Prism 9 (GraphPad Software, Boston, MA, USA). Differences in means between CON, 4FO, and 4CO were assessed using one-way analysis of variance (ANOVA) with Tukey’s post hoc test, in which differences were considered statistically significant at *p* < 0.05.

## 3. Results

### 3.1. Total Monomeric Anthocyanin Content

The total anthocyanin content of BRAE and 4FO was determined to be 240.6 mg/g of extract and 127.3 mg/g of bread in C3G equivalents, respectively. The distribution of major black rice anthocyanin species in BRAE and 4FO were also quantified and presented in Appendix B (Table A2). C3G was found to be the primary anthocyanin in BRAE, making up 211.2 mg/g BRAE, in line with previous studies [3,16]. Based on the concentration of BRAE incorporated into 4FO, the latter was expected to have approximately 624.3 mg in C3G equivalents. Thermal processing of 4FO had drastically decreased the total anthocyanin present, of which an anthocyanin retention rate of 20% was observed.

### 3.2. Effects of BRAE Fortification and Co-Digestion on Bread Digestibility

**Figure A1:**
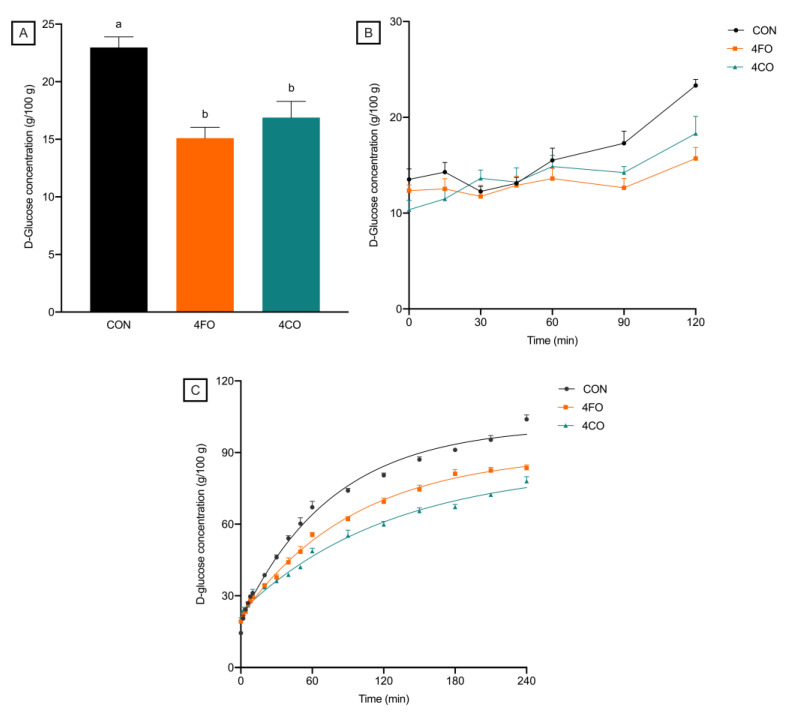
Simulated digestion profiles of bread digestibility in BRAE fortification and co-digestion experimental models. (**A**) Oral phase; (**B**) gastric phase; (**C**) intestinal phase. CON: control (wheat bread); 4FO: bread fortified with 4% BRAE (per 100 g of flour); 4CO: bread co-digested with BRAE (anthocyanin content equivalent to 4FO). Values are represented as means and standard error of means.

Overall reductions in bread digestibility were observed with BRAE usage, as exhibited by their starch digestibility profiles (Appendix C, Figure A1). Relative to CON, oral digestibility, as assessed by mean D-glucose concentrations, was significantly reduced by 34.3% and 26.5% in 4FO and 4CO, respectively (Table 1). Likewise, intestinal digestibility as assessed by IAUC was significantly reduced by 25.9% and 39.3% in 4FO and 4CO, respectively (Table 1). Reductions in the fitted kinetic constant (*k*) for intestinal starch hydrolysis were statistically significant in 4CO, but not so in 4FO (Table 1). Between 4FO and 4CO, the latter had consistently exhibited reduced parameters of intestinal digestibility. Taken together, our findings confirm the suppressive effects of BRAE on the rate of starch hydrolysis and overall starch digestibility. Differences observed between fortification and co-digestion experimental models also highlight the interference from the bread matrix on both the rate and overall bread digestibility.

### 3.3. Simulated Gastrointestinal Accessibility of Anthocyanin

**Figure 1:**
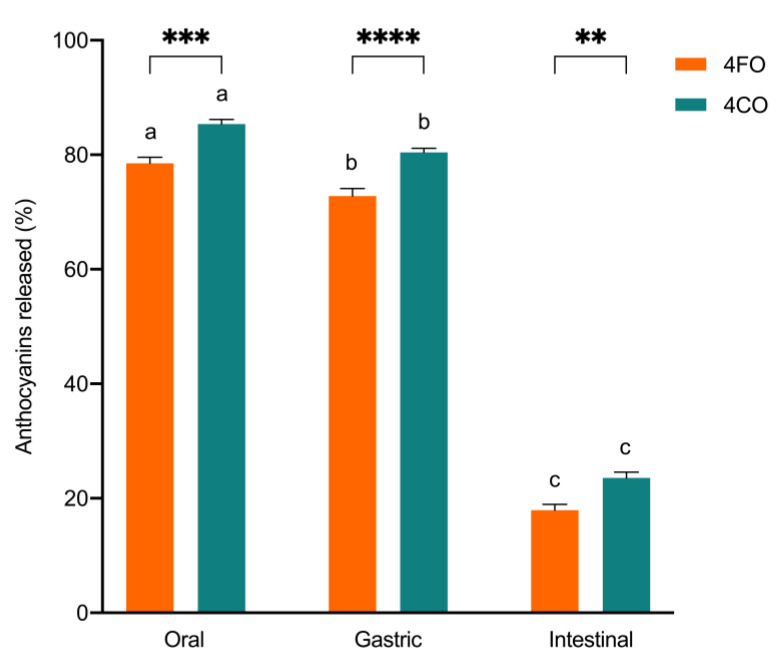
Total accessible anthocyanins after each gastrointestinal digestion phase, expressed as a percentage of initial total anthocyanins present in the corresponding experimental models. 4FO: bread fortified with 4% BRAE (per 100 g of flour); 4CO: bread co-digested with BRAE (anthocyanin content equivalent to 4FO). Values are represented as means and standard error of means. Statistical differences within digestion phases are represented by asterisks. Statistical differences within experimental models across digestion phases are represented by different lowercase letters.

**Figure 2:**
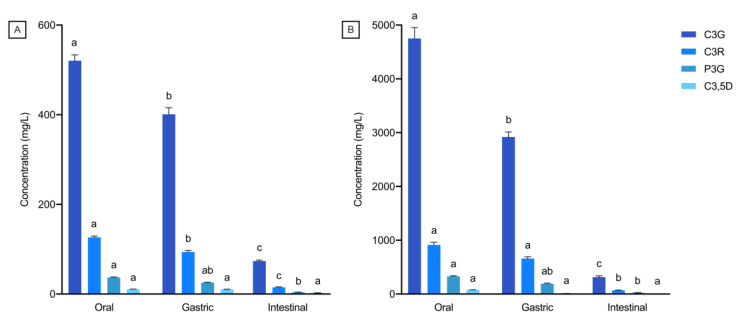
Distribution of major anthocyanin species after each gastrointestinal digestion phase. (**A**) 4FO, and (**B**) 4CO. 4FO: bread fortified with 4% BRAE (per 100 g of flour); 4CO: bread co-digested with BRAE (anthocyanin content equivalent to 4FO); C3G: cyanidin-3-glucoside; C3R: cyanidin-3-rutinoside; P3G: peonidin-3-glucoside; C3,5D: cyanidin-3,5-diglucoside. Values are represented as means and standard error of means. Statistical differences within the same species across digestion phases are represented by different lowercase letters.

The overall accessibility of black rice anthocyanins in 4FO and 4CO from each phase of bread digestion is represented in Figure 1. A general decrease in overall anthocyanin accessibility through gastrointestinal digestion was observed, with anthocyanins in 4CO being more accessible than in 4FO (Figure 1). Total anthocyanin accessibility was highest after oral digestion, with about 80% to 85% of free black rice anthocyanins detected in the digesta (Figure 1). There was a slight but statistically significant reduction in overall anthocyanin accessibility after the gastric phase in comparison to post-oral digestion. In contrast, major reductions in accessible anthocyanins were observed after intestinal digestion, with about 18% to 23% of anthocyanins detected in 4FO and 4CO, respectively (Figure 1). Among the accessible anthocyanins in 4FO and 4CO, C3G was the predominant species throughout gastrointestinal digestion (Figure 2). The accessibility of major black rice anthocyanin species reduced as digestion progressed, with no changes in their distributions across digestion phases (Figure 2). Similar trends in gastrointestinal accessibility were noted in C3G, C3R, and P3G after intestinal digestion of both 4FO and 4CO. Between 4FO and 4CO, the accessibility of the major anthocyanin species investigated across digestion phases was consistently higher in the latter experimental model (Figure 2).

The relative changes in anthocyanin content between adjacent digestion phases were also assessed and presented in Table 2. Between digestion phases, gastrointestinal changes in anthocyanin accessibility were distinctly greater when the digesta passes into the intestinal phase, as compared to the gastric phase (Table 2). On the other hand, when assessing the influence of a food matrix, reductions in anthocyanin accessibility for 4CO were generally greater than 4FO across digestion phases (Table 2). Collectively, our findings are suggestive of the influence different stages of gastrointestinal digestion have on anthocyanin accessibility. In addition, the differences between 4FO and 4CO presented here indicate the role of food matrices on the overall and gastrointestinal changes in anthocyanin accessibility.

### 3.4. Interactions between Anthocyanins and Constituents of Bread Digestion Matrices

**Figure 3:**
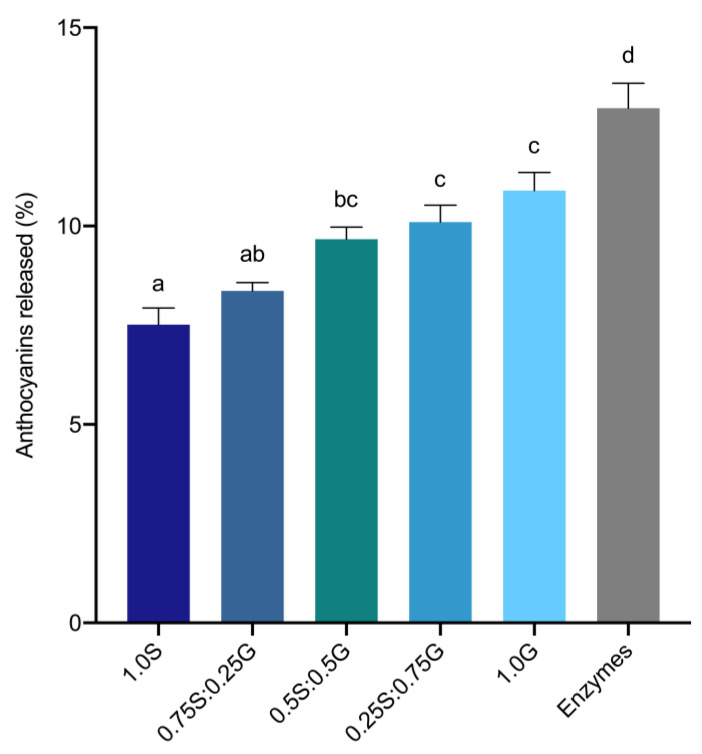
Total accessible anthocyanins from matrices of varying starch and gluten distributions, expressed as a percentage of initial total anthocyanins in the corresponding matrices. S: starch; G: gluten. Values are represented as means and standard error of means. Statistical differences in accessible anthocyanins across different matrices are represented by different lowercase letters.

Accessible black rice anthocyanins from gastrointestinal mixtures of wheat starch, gluten, or digestive enzymes were quantified to assess the interactions between anthocyanins and components in digestion matrices. Distributions of wheat starch and gluten are represented in Appendix D (Table A4). Overall accessibility of anthocyanins was observed to significantly increase with a shift towards high-protein matrices (Figure 3). When black rice anthocyanins were mixed with the different starch-gluten mixtures, 3.4% more anthocyanins were released from the gluten (1.0G) mixture in comparison to the starch mixture (1.0S). Wheat bread, with a starch-gluten distribution similar to that of 0.75S:0.25G, had 8.4% of anthocyanins released into the gastrointestinal mixture. When mixed with gastrointestinal enzymes, 13% of total anthocyanins added had been detected, which was 5.5% and 2.1% more accessible than in the starch and gluten mixtures, respectively. Altogether, it is evident that black rice anthocyanins are less accessible in starch-rich foods such as wheat bread, with accessibility improving as food matrices shift toward a higher protein content. Our findings also suggest preferential interactions of black rice anthocyanins for different constituents in food matrices and digestive enzymes during digestion, which will influence the gastrointestinal accessibility of anthocyanins.

## 4. Discussion

This work was primarily designed to investigate the effects of bread matrices on the modulatory effects of BRAE in starch digestibility. In part, we sought to understand how the natural complexities of food matrices influenced the accessibility of anthocyanins during gastrointestinal digestion, in relation to overall starch digestibility. Incorporating phytochemicals into our daily diets has been encouraged as a dietary strategy against metabolic complications [17], such as postprandial hyperglycaemia. Despite a general understanding of the benefits of anthocyanin intake, there still has been limited validation of the modulatory effects of anthocyanins specifically in the presence of food matrices commonly found in our diets, where there is the most translational relevance. As such, we simulated in this design, the digestion of two common scenarios of which anthocyanins can be consumed—through foods that have been fortified with anthocyanins, or through the co-ingestion of anthocyanins with foods.

In this study, oral and intestinal starch digestibility profiles had been significantly reduced with BRAE usage (Table 1), and this may be explained by the inhibitory effects on carbohydrases by anthocyanins. The major anthocyanins of black rice—cyanidin-3-glucoside, cyanidin-3-rutinoside, peonidin-3-glucoside, and cyanidin-3,5-diglucoside—were confirmed to be potent and synergistic inhibitors against intestinal glucosidases and pancreatic amylases [4,18]. Our findings corroborate with those of similar study designs which utilised food matrix models, noting dose-dependent reductions in starch digestibility and attributing their observations to the carbohydrase-inhibitory mechanisms of anthocyanins [5,6,19].

Findings from this study also highlighted several differences in starch digestibility and anthocyanin accessibility between digestion models, in which we collectively attribute to food matrix effects. Generically, food matrices are complex assemblies of nutrients and non-nutrients which contribute to the natural microstructures in food through physical and chemical interactions [20]. Interactions within these spatial domains (i.e., food matrix effects) have been reported to play major roles in the digestion and accessibility of nutrients [8,20,21], resulting in changes to nutrient functionalities from a food matrix in comparison to those exhibited by its free form. Additionally, food matrices may serve as a physical barrier to delay or prevent interactions between food matrix constituents, anthocyanins, and digestive enzymes [7,21]. This structural function was supported by Jenkins et al. [22], who reported that the gluten network structure, but not gluten, around starch granules in bread was responsible for retarding overall starch digestibility. Altogether, the different extents of digestion (Table 1) and anthocyanin accessibility (Figure 1) indicate that the modulatory effects of free anthocyanins on enzymatic starch hydrolysis are influenced by the presence of food matrix effects. Our findings corroborate with other reports of interactions between polyphenols and food constituents [23,24], highlighting the major role of food matrices in polyphenolic accessibility and enzyme activity, in relation to starch digestibility.

While food matrix effects do limit the free anthocyanins available to suppress starch digestion through enzyme inhibitory mechanisms, the rate and extent of starch digestibility may also be modulated by interactions between anthocyanins and starch granules. There has been limited evidence on actual mechanisms involved, but existing studies have highlighted possible non-enzymatic inhibition pathways. Kan, Oliviero, Verkerk, Fogliano, and Capuano [23] suggested the adsorption of polyphenols onto the surfaces of starch granules to sterically shield carbohydrases from their substrates. On the other hand, polyphenols were also proposed to alter the physicochemical and microstructural properties of starch through hydrogen and hydrophobic interactions, thereby producing complexes that are more resistant to enzymatic hydrolysis [25]. Although not investigated in this study, our previous work also reconciled the inter-relationship among anthocyanin functionality, alterations in starch microstructural properties, and the observed reduction in starch digestibility with anthocyanins [5]. However, it remains a challenge to exactly determine the extent to which enzymatic and non-enzymatic inhibitory pathways each contribute to the overall suppression of starch digestibility. Regardless of the possible non-enzymatic inhibitory pathways of starch digestion, it had been suggested that these inhibitory effects may still largely be dependent on the availability of free anthocyanins in the digestion matrix [23]. Taking our findings from Table 1 and Figure 1, this suggestion is co-supported by the greater extent of overall intestinal starch digestibility (Table 1) and the greater anthocyanin accessibility (Figure 1) in 4CO, relative to 4FO. Given that the total anthocyanins used in both digestion models were comparable, we also demonstrate that the co-digestion model (4CO) had a greater inhibitory effect in comparison to the fortification model (4FO), confirming the greater importance of anthocyanin accessibility in suppressing starch digestibility.

**Figure 4:**
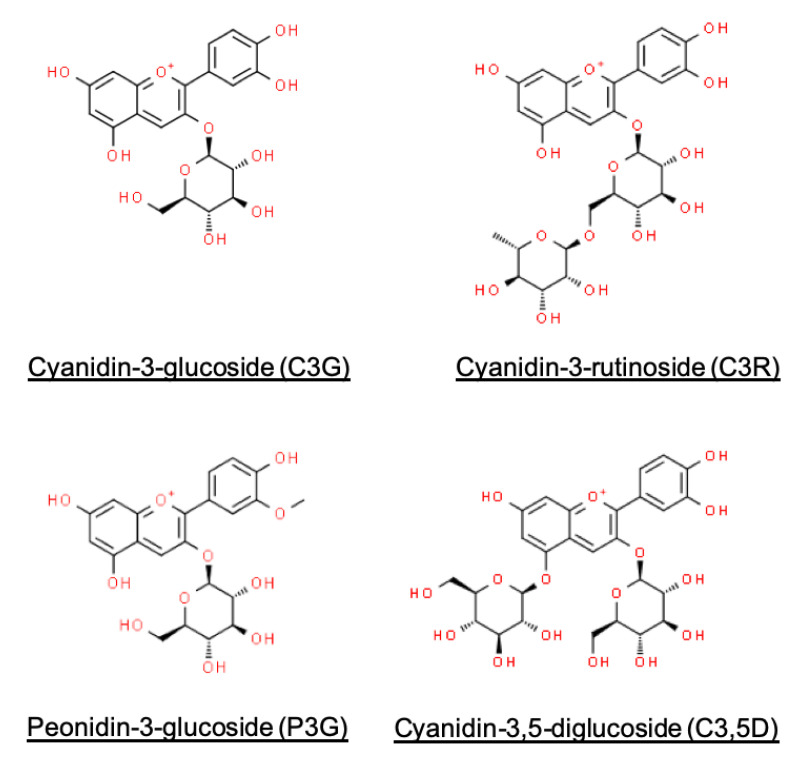
Chemical structures of major black rice anthocyanins.

On top of the possible inhibitory pathways of starch digestibility, we further explored other factors determining the extent of anthocyanin accessibility in a gastrointestinal environment. From our results, the gastrointestinal transit of food had an impact on anthocyanin accessibility. By quantifying changes in the release of anthocyanins between adjacent digestion phases, we noted a strong influence of changing pH conditions on the accessibility of anthocyanins during gastrointestinal passage (Figure 1 and Figure 2, Table 2), indicating the pH-dependence of anthocyanin stability. Findings to date confirm the instability of anthocyanins at alkaline pH conditions of the intestinal phase [2,26], in which the conversion of anthocyanins to other phenolic compounds takes place [2,9]. The complexation of anthocyanins to proteins and bile salts was also suggested, which results in the precipitation of these indigestible anthocyanin-complexes [27,28]. Differences in anthocyanin gastrointestinal stability may be due to several factors. It was reported that greater intestinal stability of anthocyanins may be attributed to oxygenated groups (e.g., -OCH_3_) in the aglycone [28], which may explain peonidin having a slightly lower loss in accessibility than cyanidin (Figure 4 and Table 2). Previous studies had also concurred with the relatively greater stability of C3,5D at intestinal pH conditions (Table 2), attributing this characteristic to the additional glycation of cyanidin (Figure 4) [2,26]. Incidentally, changes in accessibility during gastrointestinal transit were also mitigated by the presence of food matrices (Table 2). This observation may be explained by the protective function of food matrices and their constituents on anthocyanins, interacting or complexing with anthocyanins to minimise its degradation in the gastrointestinal environment [29,30].

While recent studies have confirmed the non-covalent interactions of anthocyanins with starch and/or proteins [5,23,31], the extents of their influence on anthocyanin accessibility remain elusive. Generally, we observed that the effects of adding gelatinized starch or gluten on anthocyanin accessibility were substantial (Figure 3). The addition of digestive enzymes at the quantities used in the in-vitro digestion model also reduced anthocyanin accessibility, albeit to a lesser extent. These observations were expected, given the strong affinity anthocyanins have for starch and proteins [5,31]. Co-digesting anthocyanins with matrices of different starch-protein distributions had noted changes in accessibility favouring gluten (Figure 3). Similarly, Sengul, Surek, and Nilufer-Erdil [9] reported an inhibiting effect of starch (uncooked and gelatinized), but a negligible effect of gluten, on the accessibility of anthocyanins. These findings suggested a preferential interaction of anthocyanins towards starch-rich food matrices.

**Figure 5:**
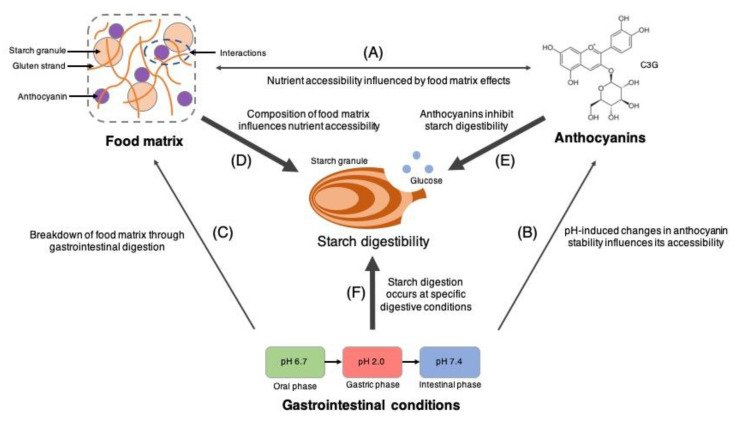
The combined influence of anthocyanins, food matrices, and gastrointestinal conditions on starch digestibility. (**A**–**C**) Inter-relationship among the factors that directly influences starch digestibility. (**D**–**F**) Factors that have a direct influence on starch digestibility.

Assessing the inter-relationship among food matrices, anthocyanin accessibility, and the gastrointestinal digestibility of starch is overtly a complex task, making it inherently difficult to attribute our observations to a singular mechanism. From our findings, it can only be generally concluded that the extent of starch digestibility and nutrient (i.e., glucose and anthocyanin) accessibility is a collective result of concurrent interactions among food matrix constituents, anthocyanins, and gastrointestinal conditions. A simplified visualisation of the roles played by the aforementioned factors in starch digestion and nutrient accessibility is presented in Figure 5. Firstly, the role of gastrointestinal conditions (i.e., pH and digestive enzymes) on the bread matrix is evident during the natural hydrolytic breakdown of starch when different gastrointestinal fluids and digestive enzymes are introduced into the digestion matrix. Changes in these conditions function to increase the accessibility of glucose available for absorption. Alterations in gastrointestinal pH have also been able to modulate the chemical stability of anthocyanins through structural modifications and redox reactions [32], which have been demonstrated to influence its gastrointestinal accessibility (Table 1 and Figure 1). Next, constituents within the bread matrix (i.e., starch and protein) and anthocyanins have been confirmed to interact non-covalently [5,23,31], inducing microstructural changes to the bread matrix [5], as well as modulating the release of anthocyanins (Figure 1 and Figure 2). Finally, the bread matrix, gastrointestinal conditions, and accessible anthocyanins function in unison to determine the overall extent of starch digestion. Starch is hydrolysed enzymatically in neutral and alkaline pH conditions of the oral and intestinal phases due to the natural optimal pH conditions of carbohydrases in humans. Accessible anthocyanins interact with carbohydrases or starch granules to inhibit bread digestion through enzymatic and non-enzymatic mechanisms, respectively. The gluten structural network in bread also functions as a natural structural barrier that mitigates interactions between starch granules, digestive enzymes, and anthocyanins [21,22]. Altogether, the numerous pathways among free anthocyanins, bread matrices, dietary components, and gastrointestinal conditions function non-exclusively and interact to determine the overall digestibility of bread.

## 5. Conclusions

This work investigated the effects of bread matrices on the modulatory effects of BRAE in relation to starch digestibility. Simulated gastrointestinal digestion of bread had not only confirmed the inhibitory effects of BRAE on bread digestibility, but also showed that the inhibition was reduced by the bread matrix. While anthocyanin accessibility was suppressed by the bread matrix and intestinal conditions, the bread matrix also had a protective effect over free anthocyanins. Notably, the observed impact of food matrix compositions on the release of anthocyanins during digestion has suggested the food matrix-specific nature of polyphenol accessibility. Conclusively, our findings highlight that the overall bread digestibility is a result of several non-exclusive interactions among black rice anthocyanins, bread matrices, its dietary constituents, and gastrointestinal conditions functioning in unison.

## Figures and Tables

**Table 1:** Fitted starch digestibility parameters for bread with (co-ingested and fortified) and without BRAE.

	Parameter	CON	4FO	4CO	*p*
Oral	C_mean_ (mmol/L)	23.0 (0.9) ^a^	15.1 (0.9) ^b^	16.9 (1.4) ^b^	0.0001
Intestinal	C_mean_ (mmol/L)	41.3 (7.1)	29.1 (5.6)	24.3 (4.6)	0.116
	AUC (mmol/L × min)	18,134 (163) ^a^	15,494 (183) ^b^	13,669 (142) ^c^	<0.0001
	IAUC (mmol/L × min)	14,681 (264) ^a^	10,877 (246) ^b^	8905 (301) ^c^	<0.0001
	*k*	0.0131 (0.0009) ^a^	0.0105 (0.0008) ^ab^	0.00777 (0.001) ^b^	0.003

C_mean_: mean concentration of D-glucose; *k*: first-order kinetic constant of fitted digestion curve; AUC: area under curve; IAUC: incremental area under curve; CON: control (wheat bread); 4FO: bread fortified with 4% BRAE (per 100 g of flour); 4CO: bread co-digested with BRAE (anthocyanin content equivalent to 4FO). Values are represented as means and standard error of means. Statistical differences between values in the same row are represented by different superscripts.

**Table 2:** Percentage reduction in accessible anthocyanins from the preceding phase of gastrointestinal digestion.

	Oral to Gastric		Gastric to Intestinal		*p*
	4FO	4CO	4FO	4CO	
Total anthocyanins (%)	10.1 (0.9) ^a^	9.8 (0.8) ^a^	68.7 (0.8) ^b^	73.4 (0.7) ^c^	<0.0001
C3G (%)	24.0 (2.6) ^a^	37.3 (2.5) ^b^	83.0 (0.7) ^c^	90.2 (0.6) ^d^	<0.0001
C3R (%)	24.2 (2.0) ^a^	25.9 (4.6) ^a^	81.5 (0.6) ^b^	90.0 (1.3) ^c^	<0.0001
P3G (%)	34.5 (1.8) ^a^	44.5 (2.8) ^b^	81.0 (1.2) ^c^	88.3 (1.1) ^d^	<0.0001
C3,5D (%)	29.2 (2.5) ^a^	58.9 (2.1) ^b^	58.8 (3.1) ^b^	69.2 (1.4) ^c^	<0.0001

4FO: bread fortified with 4% BRAE (per 100 g of flour); 4CO: bread co-digested with BRAE (anthocyanin content equivalent to 4FO); C3G: cyanidin-3-glucoside; C3R: cyanidin-3-rutinoside; P3G: peonidin-3-glucoside; C3,5D: cyanidin-3,5-diglucoside. Values are represented as means and standard error of means. Statistical differences between values in the same row are represented by different superscripts.

## Data Availability

The data presented in this study are available in the appendices.

## Appendix A. Preparation of Reagents for the Simulated Digestion Model

**Table A1:** Composition of simulated digestion fluids (SF).

For 1000 mL of Each of SF								
Stock Solution	Stock Concentration		SSF (pH 7)		SGF (pH 3)		SIF (pH 7)	
			Volume of Stock Added	Final Concentration	Volume of Stock Added	Final Concentration	Volume of Stock Added	Final Concentration
	(g/L)	(M)	(mL)	(mM)	(mL)	(mM)	(mL)	(mM)
KCl	37.3	0.5	37.75	15.1	17.25	6.9	17	6.8
KH_2_PO_4_	68	0.5	9.25	3.7	2.25	0.9	2	0.8
NaHCO_3_	84	1	17	13.6	31.25	25	106.25	85
NaCl	117	2	0	0	29.5	47.2	24	38.4
MgCl_2_(H_2_O)_6_	30.5	0.15	1.25	0.15	1	0.12	2.75	0.33
(NH4)_2_CO_3_	48	0.5	0.15	0.06	1.25	0.5	0	0
CaCl_2_(H_2_O)_2_	44.1	0.3	0.0625	1.5	0.0125	0.15	0.1	0.6
HCl		6	0.225	1.1	3.25	15.6	1.75	8.4

SF: simulated fluid; SSF: simulated salivary fluid; SGF: simulated gastric fluid; SIF: simulated intestinal fluid.

**Table A2:** Usage of reagents in each phase of the simulated gastrointestinal digestion.

	Oral Phase (pH 7)	Gastric Phase (pH 3)		Intestinal Phase (pH 7)
Sample (g)	2	4		8
Simulation fluid (μL)	1300	2950		4510
0.3M CaCl_2_ (μL)	10	2		16
Enzyme	α-amylase from porcine pancreas	Pepsin from porcine pancreas	Pancreatin from porcine pancreas	α-amyloglucosidase from *Aspergillus niger*
Final enzyme activity (U/mL)	75	2000	100	1.3
Volume of 6M HCl/NaOH	Added to adjust to pH 7	Added to adjust to pH 3		Added to adjust to pH 7
Volume of water	Top up to total volume	Top up to total volume		Top up to total volume
Total volume (mL)	4	8		16

## Appendix B. Quantification of Anthocyanin Content

**Table A3:** Monomeric anthocyanin content of BRAE and 4FO.

	BRAE		4FO	
		Incorporated into Dough	Post-Baking	Retention
	(mg/g Extract)	(mg/g Dough)	(mg/g Bread)	(%)
TMAC (C3G equivalents)	240.6 (4)	624.3 (7)	127.3 (1)	20.4
C3G	211.2 (6)	546.8 (3)	112.9 (3)	20.6
C3R	25.5 (0.2)	66.4 (1.7)	21.2 (0.5)	31.9
P3G	7.0 (0.1)	18.3 (0.5)	3.7 (0.2)	20.3
C3,5D	0.9 (0.1)	2.2 (0.1)	0.5 (0.0)	23.7

BRAE: black rice anthocyanin extract; 4FO: bread fortified with BRAE at 4% concentration; TMAC: total monomeric anthocyanin content, represented in cyanidin-3-glucoside equivalents; C3G: cyanidin-3-glucoside; C3R: cyanidin-3-rutinoside; P3G: peonidin-3-glucoside; C35D: cyanidin-3,5-diglucoside. Values are represented as means and standard error of means.

## Appendix D. Effects of Interactions between Anthocyanins and Digestion Matrix Constituents on Anthocyanin Accessibility

**Table A4:** Distribution of BRAE, wheat starch and gluten.

Matrix	Starch (g)	Gluten (g)
1.0S	3.0	0
0.75S:0.25G	2.25	0.75
0.5S:0.5G	1.5	1.5
0.25S:0.75G	0.75	2.25
1.0G	0	3.0

S: wheat starch; G: gluten.
